# *N*′-(2-Furylmethyl­ene)acetohydrazide

**DOI:** 10.1107/S1600536809033571

**Published:** 2009-08-29

**Authors:** Lu-Ping Lv, Tie-Ming Yu, Wen-Bo Yu, Wei-Wei Li, Xian-Chao Hu

**Affiliations:** a Department of Chemical Engineering, Hangzhou Vocational and Technical College, Hangzhou 310018, People’s Republic of China; b Research Center of Analysis and Measurement, Zhejiang University of Technology, Hangzhou 310014, People’s Republic of China

## Abstract

In the title mol­ecule, C_7_H_8_N_2_O_2_, the acetohydrazide group is planar within 0.014 (2) Å and forms a dihedral angle of 5.35 (8)° with the furan ring. The mol­ecule adopts a *trans* configuration with respect to the C=N bond. In the crystal, molecules are linked into a chain along the *a* axis by N—H⋯O hydrogen bonds.

## Related literature

For general background to Schiff bases, see: Cimerman *et al.* (1997 ▶); Offe *et al.* (1952 ▶); Richardson *et al.* (1988 ▶). For related structures, see: Li & Jian (2008 ▶); Tamboura *et al.* (2009 ▶).
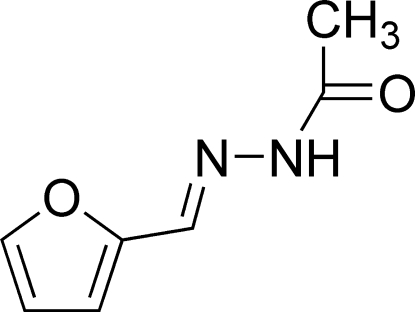


## Experimental

### 

#### Crystal data

C_7_H_8_N_2_O_2_
*M* *_r_* = 152.15
Triclinic, 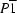

*a* = 4.4618 (13) Å
*b* = 9.275 (3) Å
*c* = 10.541 (4) Å
α = 112.069 (15)°
β = 98.135 (16)°
γ = 101.945 (11)°
*V* = 383.8 (2) Å^3^
*Z* = 2
Mo *K*α radiation
μ = 0.10 mm^−1^
*T* = 223 K
0.19 × 0.17 × 0.16 mm

#### Data collection

Bruker SMART CCD area-detector diffractometer
Absorption correction: multi-scan (*SADABS*; Bruker, 2002 ▶) *T*_min_ = 0.978, *T*_max_ = 0.982
2182 measured reflections
1400 independent reflections
918 reflections with *I* > 2σ(*I*)
*R*_int_ = 0.023

#### Refinement

*R*[*F* ^2^ > 2σ(*F* ^2^)] = 0.051
*wR*(*F* ^2^) = 0.174
*S* = 0.95
1400 reflections
101 parameters
H-atom parameters constrained
Δρ_max_ = 0.19 e Å^−3^
Δρ_min_ = −0.18 e Å^−3^

### 

Data collection: *SMART* (Bruker, 2002 ▶); cell refinement: *SAINT* (Bruker, 2002 ▶); data reduction: *SAINT*; program(s) used to solve structure: *SHELXS97* (Sheldrick, 2008 ▶); program(s) used to refine structure: *SHELXL97* (Sheldrick, 2008 ▶); molecular graphics: *SHELXTL* (Sheldrick, 2008 ▶); software used to prepare material for publication: *SHELXTL*.

## Supplementary Material

Crystal structure: contains datablocks I, global. DOI: 10.1107/S1600536809033571/bg2291sup1.cif

Structure factors: contains datablocks I. DOI: 10.1107/S1600536809033571/bg2291Isup2.hkl

Additional supplementary materials:  crystallographic information; 3D view; checkCIF report

## Figures and Tables

**Table 1:** Hydrogen-bond geometry (Å, °)

*D*—H⋯*A*	*D*—H	H⋯*A*	*D*⋯*A*	*D*—H⋯*A*
N2—H2*A*⋯O2^i^	0.86	2.06	2.904 (3)	167

Symmetry code: (i) 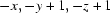.

**Fig. 1.**
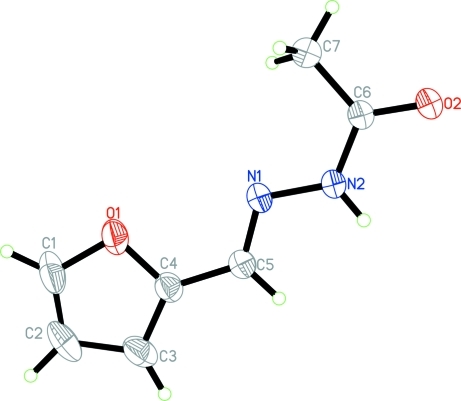
The molecular structure of the title compound. Displacement ellipsoids are drawn at the 40% probability level.

**Fig. 2.**
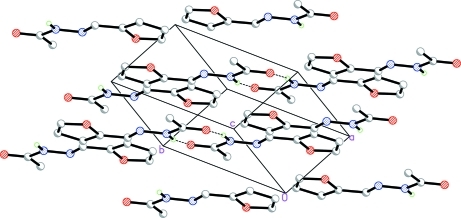
Part of the crystal packing of the title compound. Hydrogen bonds are shown as dashed lines.

## supplementary crystallographic information

### Comment

Schiff bases have attracted much attention due to the possibility of their analytical applications (Cimerman *et al.*, 1997). They are also important ligands, which have been reported to have mild bacteriostatic activity and are used as potential oral iron-chelating drugs for genetic disorders such as thalassemia (Offe *et al.*, 1952; Richardson *et al.*, 1988). Metal complexes based on Schiff bases have received considerable attention because they can be utilized as model compounds of active centres in various complexes (Tamboura *et al.*, 2009). We report here the crystal structure of the title compound (Fig. 1).

The acetohydrazide group is planar and it forms a dihedral angle of 5.35 (8)° with the benzene ring. The molecule adopts a *trans* configuration with respect to the C═N bond. Bond lengths and angles are comparable to those observed for *N*'-[1-(4-methoxyphenyl)ethylidene]acetohydrazide (Li *et al.*, 2008).

The molecules are linked into a chain along the *a* axis by N—H···O hydrogen bonds (Table 1, Fig.2).

### Experimental

Furfuraldehyde (0.96 g, 0.01 mol) and acetohydrazide (0.74 g, 0.01 mol) were dissolved in stirred methanol (20 ml) and left for 1.5 h at room temperature. The resulting solid was filtered off and recrystallized from ethanol to give the title compound in 87% yield. Single crystals suitable for X-ray analysis were obtained by slow evaporation of an ethanol solution at room temperature (m.p. 485–487 K).

### Refinement

H atoms were positioned geometrically (N-H = 0.86 Å and C-H = 0.93 or 0.96Å) and refined using a riding model, with *U*_iso_(H) = 1.2*U*_eq_(C,N) and 1.5*U*_eq_(C_methyl_).

### Crystal data

**Table:**

C_7_H_8_N_2_O_2_	*Z* = 2
*M**_r_* = 152.15	*F*(000) = 160
Triclinic, *P*1	*D*_x_ = 1.317 Mg m^−^^3^
Hall symbol: -P 1	Mo *K*α radiation, λ = 0.71073 Å
*a* = 4.4618 (13) Å	Cell parameters from 1400 reflections
*b* = 9.275 (3) Å	θ = 2.2–25.5°
*c* = 10.541 (4) Å	µ = 0.10 mm^−^^1^
α = 112.069 (15)°	*T* = 223 K
β = 98.135 (16)°	Block, colourless
γ = 101.945 (11)°	0.19 × 0.17 × 0.16 mm
*V* = 383.8 (2) Å^3^	

### Data collection

**Table:**

Bruker SMART CCD area-detector diffractometer	1400 independent reflections
Radiation source: fine-focus sealed tube	918 reflections with *I* > 2σ(*I*)
graphite	*R*_int_ = 0.023
φ and ω scans	θ_max_ = 25.5°, θ_min_ = 2.2°
Absorption correction: multi-scan (*SADABS*; Bruker, 2002)	*h* = −5→5
*T*_min_ = 0.978, *T*_max_ = 0.982	*k* = −11→11
2182 measured reflections	*l* = −12→12

### Refinement

**Table:**

Refinement on *F*^2^	Primary atom site location: structure-invariant direct methods
Least-squares matrix: full	Secondary atom site location: difference Fourier map
*R*[*F*^2^ > 2σ(*F*^2^)] = 0.051	Hydrogen site location: inferred from neighbouring sites
*wR*(*F*^2^) = 0.174	H-atom parameters constrained
*S* = 0.95	*w* = 1/[σ^2^(*F*_o_^2^) + (0.1191*P*)^2^] where *P* = (*F*_o_^2^ + 2*F*_c_^2^)/3
1400 reflections	(Δ/σ)_max_ < 0.001
101 parameters	Δρ_max_ = 0.19 e Å^−^^3^
0 restraints	Δρ_min_ = −0.18 e Å^−^^3^

### Special details

**Table:**

Geometry. All esds (except the esd in the dihedral angle between two l.s. planes) are estimated using the full covariance matrix. The cell esds are taken into account individually in the estimation of esds in distances, angles and torsion angles; correlations between esds in cell parameters are only used when they are defined by crystal symmetry. An approximate (isotropic) treatment of cell esds is used for estimating esds involving l.s. planes.
Refinement. Refinement of F^2^ against ALL reflections. The weighted R-factor wR and goodness of fit S are based on F^2^, conventional R-factors R are based on F, with F set to zero for negative F^2^. The threshold expression of F^2^ > 2sigma(F^2^) is used only for calculating R-factors(gt) etc. and is not relevant to the choice of reflections for refinement. R-factors based on F^2^ are statistically about twice as large as those based on F, and R- factors based on ALL data will be even larger.

### Fractional atomic coordinates and isotropic or equivalent isotropic displacement parameters (Å^2^)

**Table:**

	*x*	*y*	*z*	*U*_iso_*/*U*_eq_	
O1	0.5605 (4)	0.02737 (18)	0.15111 (17)	0.0701 (6)	
O2	−0.0574 (4)	0.58652 (17)	0.37154 (16)	0.0608 (5)	
C6	0.0623 (5)	0.4844 (2)	0.3031 (2)	0.0495 (6)	
C5	0.4180 (5)	0.2017 (2)	0.3531 (2)	0.0505 (6)	
H5	0.4064	0.2313	0.4465	0.061*	
C4	0.5534 (5)	0.0730 (3)	0.2899 (2)	0.0518 (6)	
C3	0.6756 (6)	−0.0219 (3)	0.3396 (3)	0.0699 (7)	
H3	0.6993	−0.0158	0.4307	0.084*	
C2	0.7615 (7)	−0.1334 (3)	0.2251 (4)	0.0826 (9)	
H2	0.8504	−0.2151	0.2268	0.099*	
C7	0.0863 (7)	0.4567 (3)	0.1567 (3)	0.0774 (8)	
H7A	0.0404	0.5436	0.1365	0.116*	
H7B	−0.0622	0.3557	0.0911	0.116*	
H7C	0.2963	0.4531	0.1481	0.116*	
C1	0.6908 (7)	−0.0983 (3)	0.1166 (3)	0.0850 (9)	
H1	0.7253	−0.1522	0.0282	0.102*	
N2	0.1808 (4)	0.39408 (19)	0.36019 (18)	0.0499 (5)	
H2A	0.1737	0.4099	0.4454	0.060*	
N1	0.3127 (4)	0.27748 (19)	0.28617 (18)	0.0487 (5)	

### Atomic displacement parameters (Å^2^)

**Table:**

	*U*^11^	*U*^22^	*U*^33^	*U*^12^	*U*^13^	*U*^23^
O1	0.0859 (12)	0.0630 (10)	0.0655 (11)	0.0438 (9)	0.0226 (9)	0.0178 (8)
O2	0.0828 (12)	0.0579 (9)	0.0577 (10)	0.0425 (9)	0.0275 (8)	0.0258 (7)
C6	0.0587 (13)	0.0462 (11)	0.0482 (12)	0.0236 (10)	0.0167 (10)	0.0186 (9)
C5	0.0532 (13)	0.0525 (12)	0.0532 (12)	0.0233 (11)	0.0170 (10)	0.0241 (10)
C4	0.0512 (13)	0.0504 (12)	0.0594 (13)	0.0210 (10)	0.0180 (10)	0.0240 (10)
C3	0.0702 (16)	0.0672 (15)	0.094 (2)	0.0355 (13)	0.0237 (14)	0.0469 (14)
C2	0.0707 (17)	0.0558 (15)	0.130 (3)	0.0367 (13)	0.0245 (17)	0.0383 (17)
C7	0.117 (2)	0.0843 (17)	0.0598 (15)	0.0623 (17)	0.0354 (15)	0.0384 (13)
C1	0.091 (2)	0.0633 (16)	0.091 (2)	0.0469 (16)	0.0227 (16)	0.0084 (14)
N2	0.0627 (12)	0.0505 (10)	0.0475 (10)	0.0313 (9)	0.0216 (9)	0.0214 (8)
N1	0.0533 (11)	0.0457 (10)	0.0530 (11)	0.0244 (8)	0.0182 (8)	0.0195 (8)

### Geometric parameters (Å, °)

**Table:**

O1—C1	1.361 (3)	C3—H3	0.9300
O1—C4	1.369 (3)	C2—C1	1.317 (4)
O2—C6	1.228 (2)	C2—H2	0.9300
C6—N2	1.347 (3)	C7—H7A	0.9600
C6—C7	1.490 (3)	C7—H7B	0.9600
C5—N1	1.276 (3)	C7—H7C	0.9600
C5—C4	1.431 (3)	C1—H1	0.9300
C5—H5	0.9300	N2—N1	1.373 (2)
C4—C3	1.345 (3)	N2—H2A	0.8600
C3—C2	1.425 (4)		
			
C1—O1—C4	106.4 (2)	C3—C2—H2	126.6
O2—C6—N2	120.26 (19)	C6—C7—H7A	109.5
O2—C6—C7	122.07 (19)	C6—C7—H7B	109.5
N2—C6—C7	117.66 (19)	H7A—C7—H7B	109.5
N1—C5—C4	122.5 (2)	C6—C7—H7C	109.5
N1—C5—H5	118.7	H7A—C7—H7C	109.5
C4—C5—H5	118.7	H7B—C7—H7C	109.5
C3—C4—O1	109.4 (2)	C2—C1—O1	111.0 (2)
C3—C4—C5	132.4 (2)	C2—C1—H1	124.5
O1—C4—C5	118.18 (19)	O1—C1—H1	124.5
C4—C3—C2	106.5 (2)	C6—N2—N1	121.83 (18)
C4—C3—H3	126.8	C6—N2—H2A	119.1
C2—C3—H3	126.8	N1—N2—H2A	119.1
C1—C2—C3	106.7 (2)	C5—N1—N2	115.38 (18)
C1—C2—H2	126.6		
			
C1—O1—C4—C3	0.0 (3)	C3—C2—C1—O1	−0.8 (3)
C1—O1—C4—C5	−178.3 (2)	C4—O1—C1—C2	0.5 (3)
N1—C5—C4—C3	−179.8 (2)	O2—C6—N2—N1	179.19 (17)
N1—C5—C4—O1	−1.9 (3)	C7—C6—N2—N1	−1.6 (3)
O1—C4—C3—C2	−0.4 (3)	C4—C5—N1—N2	177.93 (17)
C5—C4—C3—C2	177.6 (2)	C6—N2—N1—C5	179.50 (19)
C4—C3—C2—C1	0.7 (3)		

### Hydrogen-bond geometry (Å, °)

**Table:**

*D*—H···*A*	*D*—H	H···*A*	*D*···*A*	*D*—H···*A*
N2—H2A···O2^i^	0.86	2.06	2.904 (3)	167

Symmetry codes: (i) −*x*, −*y*+1, −*z*+1.
